# Effect of Human Disturbance on Feeding Behavior and Activity Time Budget of Lesser Adjutant Stork *Leptoptilos javanicus* (Horsfield, 1821) in Nepal

**DOI:** 10.1002/ece3.71643

**Published:** 2025-06-27

**Authors:** Santosh Bajagain, Jhamak Bahadur Karki, Yajna Prasad Timilsina, Menuka Maharjan, Aavas Pradhan, Nabaraj Pudasaini, Prashant Rokka, Surendra Maharjan

**Affiliations:** ^1^ Bird Conservation Nepal Kathmandu Nepal; ^2^ School of Forestry and Natural Resource Management, Bird Conservation Nepal Kathmandu Nepal; ^3^ Kathmandu Forestry College Kathmandu Nepal; ^4^ Institute of Forestry, Tribhuvan University Pokhara Kaski Nepal; ^5^ School of Forestry and Natural Resource Management, Institute of Forestry, Tribhuvan University Hetauda Makwanpur Nepal; ^6^ Ministry of Forests and Environment Kathmandu Nepal; ^7^ Faculty of Forestry, Agriculture and Forestry University Hetauda Makwanpur Nepal; ^8^ Chapman University Orange California USA

**Keywords:** anthropogenic disturbance, avian ecology, behavior, diurnal activity, vigilance

## Abstract

Understanding how animals allocate their time among essential behaviors such as foraging, self‐maintenance, and reproduction is critical for effective conservation, especially in human‐modified landscapes. The study investigated how Lesser Adjutant Storks ( *Leptoptilos javanicus* ) adjust their behavior in response to human disturbance in the Janakinagar‐Murtiya Important Bird and Biodiversity Area of Nepal. Using the Focal Animal Sampling method, we captured 600 min of video footage along road transects during the summer of 2023 and winter of 2024. The analysis revealed significant seasonal shifts. During the summer, vigilance dominated the activity budget (47.18%), while feeding was comparatively low (15.06%). In contrast, during winter, storks prioritized feeding (30.33%) over vigilance (23.32%). Kruskal–Wallis and Dunn's post hoc tests confirmed that both feeding and vigilance varied significantly across seasons (*p* < 0.05), suggesting that species adjust their time budgets based on resource availability and predation risk. Moreover, multinomial regression analysis indicated that human disturbance significantly influenced vigilance, with storks in high disturbance areas displaying greater vigilance than those in low disturbance areas (*p* < 0.05). These findings were further supported by a Likelihood Ratio Test (LRT = 263.82, df = 6, *p* < 0.05), confirming that disturbance level had a significant effect on behavioral variation. Collectively, the study suggests that vigilance behavior increases in both frequency and intensity under higher disturbance, potentially at the cost of feeding time and energy intake. This underscores the importance of minimizing human disturbance and conserving critical foraging habitats to support the long‐term survival of the Lesser Adjutant Stork population in increasingly human‐dominated landscapes.

## Graphical abstract

The study presents the activity budget of Lesser Adjutant Stork ( *Leptoptilos javanicus* ) as well as its behavior against human disturbance in summer and winter season. The study conducted in Janakinagar‐Murtiya Important Bird and Biodiversity Area of lowland Nepal highlights how bird species living in human‐dominated landscape adjust their behavioral pattern as well as respond to human disturbance.

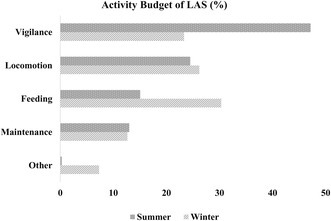

## 1 Introduction

There are 20 species of storks in the Ciconiidae family of birds, eight of which are found in Nepal (Gula et al. 2023). Nepal is home to two species of adjutant storks: the Greater Adjutant Stork, which is possibly extinct in the country, and the Lesser Adjutant Stork, which is a local resident species (Birdlife International 2024). The Lesser Adjutant Stork (LAS hereafter) ( *Leptoptilos javanicus* ) is designated as Vulnerable in Nepal's Red Data Book (Inskipp et al. 2016), with global population estimates ranging from 5000 to 15,000 individuals, and 200 to 700 individuals in Nepal (Inskipp et al. 2016; Birdlife International 2024). LAS, a large aquatic bird, resides in diverse wetland habitats, favoring tall, mature trees like the Silk‐cotton Tree ( *Bombax ceiba* ) and Kadam (*Adina cordifolia*) for nesting. However, its survival is jeopardized by various human activities such as hunting, illegal tree cutting, disturbance, and collection of LAS prey by people as bushmeat (Bhattarai et al. 2021; Katuwal et al. 2022). Human disruptions, akin to predation risks, can disrupt stork behavior, prompting increased vigilance and diminished foraging time (Frid and Dill 2002). According to the risk‐disturbance hypothesis, human disturbances might have a more pronounced impact on wildlife behavior than natural predators (Frid and Dill 2002). Understanding stork activity‐time patterns and their response to human interference is pivotal for shaping effective conservation strategies. Activity‐time patterns reflect ecological necessities and external pressures, aiding in the formulation of conservation plans (Paulus 1984; Das et al. 2011).

The term “activity budget” refers to the time allocation of individual animals to various activities, including foraging, self‐maintenance, and breeding (Das et al. 2011). It provides quantitative insights into how animals spend their time, influenced by environmental conditions and species‐specific adaptations (Migoya et al. 1994; Willmer et al. 2009). Activity budgets are crucial for understanding behavioral ecology as they directly impact metabolism and energy requirements (Halle and Stenseth 2012), reflecting a species' physical habitat and environmental conditions (Paulus 1984; Datta 2014).

Wetland birds, with their specialized adaptations to aquatic environments and observable behaviors, are ideal for studying activity budgets, as their time allocation to various activities like foraging, resting, and preening provides valuable insights into their ecological roles and adaptations (Morrier and McNeil 1991). Diurnal activity budgets of wetland birds vary widely among species, individuals, and seasons, influenced by factors like climate, food availability, and habitat types (Turner 1982; Yang et al. 2007). Foraging patterns of wetland birds also provide insights into ecosystem health and can indicate habitat quality (Gopi Sundar and Kittur 2012). Birds spend less time foraging in high‐quality environments, leading to higher breeding success, suggesting a strategic decision‐making process in time allocation (Janiszewski et al. 2014; Osborn et al. 2017; Haig et al. 2019). Understanding foraging strategies is critical for wetland bird behavioral ecology (Sommerfeld et al. 2013), necessitating an examination of factors influencing these strategies (Whitehorne 2010).

There is global concern for the conservation of LAS due to various threats including farmland conversion, wetland degradation, pesticide use, and food scarcity (Subba et al. 2008; Bajagain et al. 2019). LAS lives close to human settlement (Karki and Thapa 2013) and this proximity to humans might influence the foraging habitats of LAS thereby compelling it to alter its behavior in response to human activities. In areas with higher disturbances, storks are expected to allocate more time to behaviors such as vigilance and locomotion, as these activities may help mitigate risks posed by disturbances. Conversely, in low disturbance environments, storks are more likely to engage in behaviors like feeding and maintenance, which are essential for energy acquisition and self‐care. Regarding seasonality, the effects may vary due to changes in resource availability and environmental conditions. For instance, during breeding season, storks may prioritize activities differently compared to nonbreeding seasons, potentially amplifying the influence of disturbances on their behavior.

Despite general concerns about human disturbances on LAS in Nepal, there is a lack of studies observing their behavioral responses to such disturbances. Conducting studies like this, which document activity budgets, can serve as reliable indicators of habitat quality and disturbance levels (Schummer and Eddleman 2003; Zimmer et al. 2011; Zha et al. 2019). Thus, this study can provide valuable insights for LAS conservation planning, offering baseline information and practical references for protection strategies.

## 2 Methods

### 2.1 Study Site and Species

**FIGURE 1:**
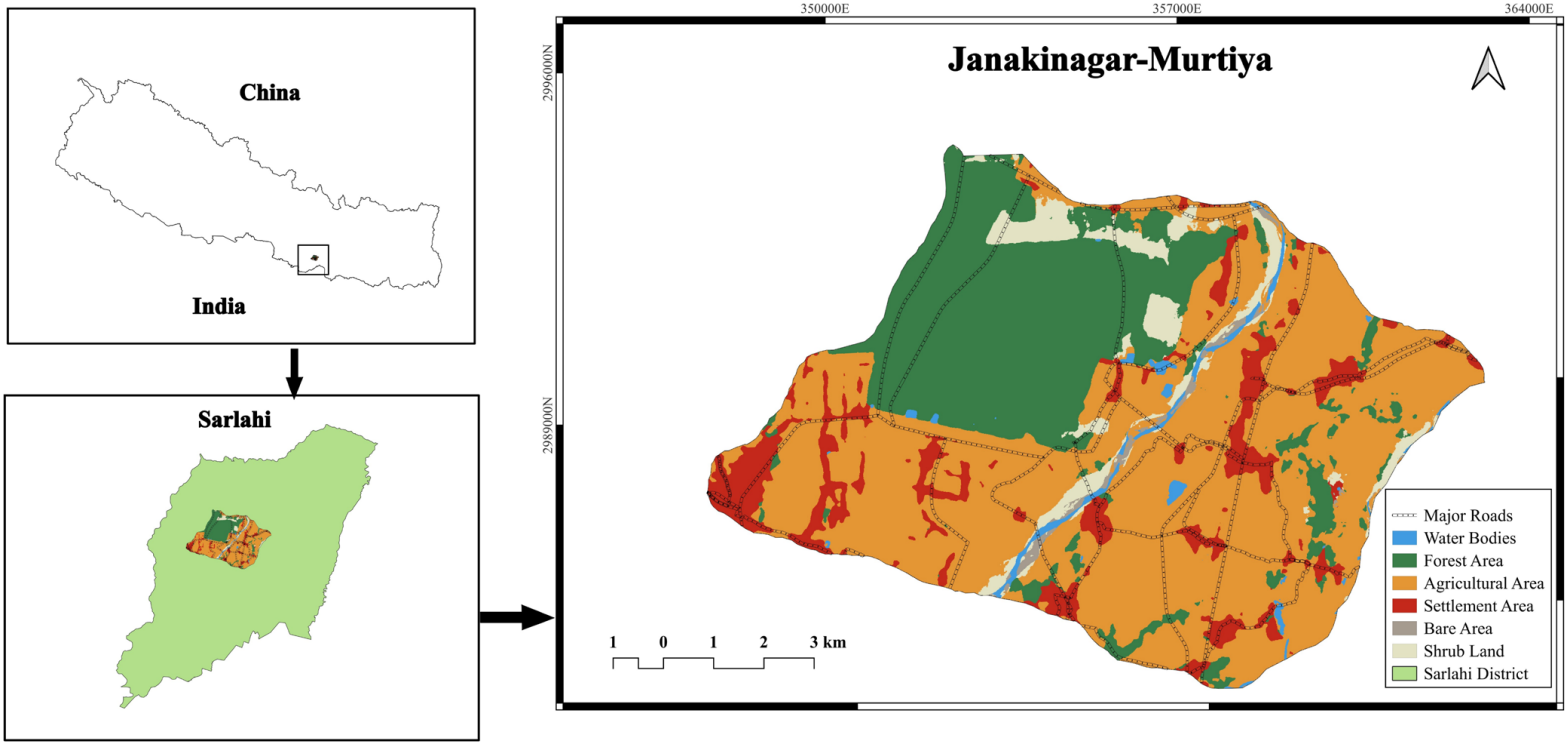
Study area map.

The research was conducted in the Janakinagar‐Murtiya Important Bird and Biodiversity Area (IBA) in the Sarlahi district of Madhesh Province (Figure 1). This area is part of the lower tropical bioclimatic zone, characterized by a subtropical monsoon climate and a tropical forest ecosystem (BPP 1995; Malla et al. 2020). The lower tropical zone, at elevations below 300 m, encompasses two primary ecosystems: the Terai tropical *Shorea robusta* forest and the Terai cultivated land (BPP 1995). Within the Janakinagar‐Murtiya IBA, there are diverse forest types, including the Janakinagar Collaborative Forest, Mukteshwor Community Forest, and Bihani Community Forest, with the Janakinagar‐Murtiya Forest being the most extensive, covering approximately 1915 ha. This natural forest is bordered by planted forests, predominantly Eucalyptus spp. The surrounding agricultural landscape provides additional habitats for birds, characterized by flat terrain, crop fields, and scattered trees such as the *Bombax ceiba* . The dominant vegetation within the forest consists of *Shorea robusta* trees. Agricultural practices in the area include the cultivation of multiple crops throughout the year. Flooded rice paddies are common during the monsoon season (June–September), maize is grown in the summer, and wheat and lentils are cultivated during the winter months (November–February). Additionally, sugarcane is cultivated year‐round in certain areas (Katuwal et al. 2020). The significant population of LAS in Janakinagar‐Murtiya qualifies it as an IBA under globally threatened criteria (Katuwal et al. 2022).

The behavior of the LAS was recorded using video observations along a road transect following the method described by (Ghimire et al. 2021). The survey was conducted between August 2023 and January 2024, with field visits made on the 15th of each month for three consecutive days, from 8:00 to 10:00 h and 14:00 to 16:00 h, the periods of peak LAS activity (Bonter et al. 2013; Ghimire et al. 2021). Surveys were conducted across wetland, farmland, grassland and forest habitats where LAS are known to forage. A Nikon Coolpix P1000 and P900 camera was used to record videos of single LAS individuals from a distance of approximately 200 m to minimize disturbance. Across 8 months, a total of 24 survey days were completed, resulting in 36,000 s (10 h) of behavioral footage.

To collect behavioral data, we used the Focal Animal Sampling Method (Altmann 1974; Zha et al. 2019), in which a focal individual was continuously observed and all visible behaviors were recorded until the bird moved out of sight. Following Ghimire et al. (2021), each focal individual was selected based on its visibility and accessibility along transects, and individuals were not repeatedly sampled within the same day and same location. However, we acknowledge that no explicit measures were implemented to avoid sampling the same individual on different survey days, consistent with the approach outlined by Ghimire et al. (2021).

The road routes were surveyed multiple times within each season. The birds were neither marked nor individually identifiable, thus to minimize the repeated sampling of the same individual, repeated surveys on the same routes were spaced at least 1 month apart. Therefore, all observations were treated as independent samples.

Regarding the differentiation of sex, the species studied exhibits limited sexual dimorphism, making it challenging to visually distinguish males from females. Furthermore, both sexes share equal roles in chick‐rearing, which reduced the necessity for sex differentiation in the context of our study objectives.

Finally, our research focused on the behavior of birds during two distinct seasons—breeding (summer) and nonbreeding (winter). To ensure consistency, we exclusively sampled adult birds during the study. At the time of our observations, chicks were not yet fully fledged, minimizing the likelihood of including juveniles in our sampling.

During our study, the behavior of LAS was categorized into five major functional categories: Locomotion, Maintenance, Vigilance, Feeding, and Other Behaviors, based on the classification framework proposed by Kahl (1972) and adapted by Ghimire et al. (2021). The total time (sec) spent in each category of behavior was obtained from video recordings. Each recording session was treated as an independent sample for analysis, and the proportion of time spent in each functional category was computed for each sample. The time spent on each behavior was initially recorded; however, to examine the effects of disturbances (such as crop harvesting, livestock grazing, fishing, vehicle and human movement) on LAS behavior, categorical variables were used instead of time‐based measurements. These categorical variables allowed us to assess the behavioral responses of LAS under varying levels of disturbance more effectively. A detailed description of each functional category and specific behaviors is provided in Table 1.

**TABLE 1:** Functional categories of LAS behavior.

Functional category	Behavior	Description
Locomotion	Walking	Bird moves with its wings closed. Neck is stretched and retracted time to time during walking.
	Leaping	Short jump where one legs is stretched first. Distance between steps is greater than walking.
	Running	Bird moves at faster pace than walking mainly during flight initiation, intrusion by potential predator or during feeding to catch prey such as snakes which moves very fast
	Flying	Short and rapid wing strokes at beginning. Wings are steady to glide once it reaches a certain height. Glide is also common before landing on ground. Bird also performs rare acrobatic movements while diving from altitude to foraging ground or roosting tree or nest.
Maintenance	Auto preening	Bird uses beak to smoothen and clean its own wings specially flanks, belly, breast and legs. Bird bends its neck to clean around its own back.
	Scratching	Bird uses one leg to scratch around the neck, head and under wings region. Bird balances the body with another leg.
	Body fluffing	Feathers on back and around breast region erects where bird hides its beak. Neck stretches rarely during this behavior.
	Wing spreading	Wings spread and bright narrow orange‐red bands on underside of the forearm are clearly visible. Bird stretches its neck and body while its tail points toward the ground. Sometimes, wings spread partially.
	Leg stretching	One of the legs stretches while another balances the body.
	Head shaking	Bird shakes its head horizontally.
	Yawning	Bird yawns usually after foraging by opening its beak. One bird of the pair followed yawning of another at two instances.
Vigilance	Stationary head	Bird stretches its neck while foraging under tall habitat cover or in wetlands. Head stretches and scans in one direction.
	Head Movement	Bird stretches its neck and moves head sideward quickly.
Feeding	Foraging	Head is down to the ground searching for prey. Neck stretches only to look for prey at distance otherwise neck retracts.
	Probing	Probing are two kinds; (1) Bird walks and probes singly at one place. (2) Bird is stationery and probes for multiple times at the same location.
	Food preparation	Prey is taken to the beak closer to tip. Bird shakes its head, slightly open its beak and maintain position of prey on the beak. Larger prey is immobilized first on ground.
	Eating	Bird lifts its head slightly and ingest the food. Smaller prey is tossed while larger are slowly ingested.
	Drinking	Bird lowers its head, dips its beak horizontally on water, uplift the head and swallow water.

The disturbance type and distance to disturbances were recorded before each behavioral session began. A number of human activities, including movement, noise, livestock grazing, and harvesting, were further classified as human disturbances. Disturbance types were categorized based on the proximity of LAS to farmers, roads or traffic, and human settlements: within 50 m was classified as high disturbance, 50–100 m as medium disturbance, and greater than 100 m as low disturbance.

A rangefinder was used to measure the distance from the observer to both the focal stork and the approaching disturbance source. The distance between the stork and the disturbance source was then calculated using basic geometry, based on these two measurements and the estimated angle between them. When the stork and disturbance source were aligned from the observer's viewpoint, the distance was approximated by subtracting one rangefinder reading from the other. If the stork moved during observation, measurements were updated to ensure accuracy.

### 2.2 Statistical Analysis

All statistical analyses were conducted in R version 4.0.0 (R Core Team, 2023). Data wrangling and filtering were performed using the dplyr package (Wickham et al. 2014), and statistical tests were carried out using base R functions and the FSA package (Ogle et al. 2015). During the behavioral observations, we recorded the time (in seconds) that individual Lesser Adjutant Storks spent on five functional behavior categories: Feeding, Vigilance, Locomotion, Maintenance, and Other. However, for the analysis of seasonal variation, we excluded the “Other” category to focus on the four primary and consistently observed behaviors.

To assess whether Lesser Adjutant Storks exhibited seasonal differences in the time spent on different behaviors, we first tested for normality of the time data using the Shapiro–Wilk test. The results indicated violations of normality assumptions, justifying the use of nonparametric methods. Accordingly, we performed separate Kruskal‐Wallis rank sum tests for each of the four main behavioral categories—vigilance, feeding, locomotion, and maintenance (excluding “other”)—to evaluate seasonal differences in time allocation (in seconds) between summer and winter. This test is suitable for comparing more than two groups when the assumptions of normality and homogeneity of variance are not met. For each behavior, the test statistic and corresponding *p* value were recorded, and significance was determined at the 0.05 level.

For behaviors that showed statistically significant differences between seasons (i.e., Feeding and Vigilance), we conducted Dunn's post hoc test using the dunnTest () function from the FSA package. A Bonferroni correction was applied to adjust for multiple comparisons and reduce the risk of false positives (Type I error). This test allowed us to determine which specific seasonal pair (Summer vs. Winter) differed significantly in time allocation for each behavior. Only the Feeding and Vigilance behaviors showed significant seasonal differences, thus the post hoc analysis was conducted exclusively for these two behaviors.

Further, to evaluate the influence of disturbance levels on the behavioral responses of storks, we employed multinomial logistic regression, appropriate for modeling categorical outcome variables with more than two levels. The dependent variable taken was the Functional Category of behavior, classified into four discrete categories: Feeding, Locomotion, Maintenance, and Vigilance excluding others. The independent variable was the Level of Disturbance, categorized as Low, Medium, or High based on direct field assessments. The regression analysis utilized only the categorical classification of behavior. Each behavioral event observed during a sampling session was treated as a single independent data point based on its category, and the corresponding time duration was excluded from the model. This approach allowed for the inclusion of all observed behavioral events while satisfying the assumptions of multinomial logistic regression, which requires a categorical dependent variable. The analysis was conducted using the multinom () function from the nnet package in R (Ripley 2009). Two models were constructed: a full model including Level of Disturbance as a predictor and a null model containing only the intercept. A Likelihood Ratio Test (LRT) was used to compare these models and assess whether the inclusion of the disturbance variable significantly improved model fit. In addition to the model comparison through the Likelihood Ratio Test (LRT), which confirmed that the multinomial logistic regression model significantly improved the fit over the null model, further validation was conducted using the Wald *Z*‐test.

The Wald *Z*‐test was applied to evaluate the statistical significance of the estimated coefficients for each predictor variable within the model. For each behavior (locomotion, maintenance, and vigilance), *Z*‐scores were computed by dividing the regression coefficients by their corresponding standard errors. These *Z*‐scores were then tested against a standard normal distribution to derive *p* values, indicating the strength of evidence against the null hypothesis (i.e., no effect).

A significant *p* value suggests that the corresponding predictor variable (disturbance levels) has a notable impact on the particular behaviors being examined. In contrast, nonsignificant *p* values indicate a weaker or no effect of the predictor on the behaviors. This method allowed for a more nuanced understanding of how each level of disturbance affects the stork's behaviors, providing insights into the key predictors that drive behavior changes in response to environmental disturbances.

### 2.3 The Models Used in the Study Are as Follows

#### 2.3.1 Null Model

(1) $$\ln\frac{\left(\pi_{j}\right)}{\left(\pi_{j}*\right)}=\beta_{0j},j\neq j^{*}$$

where, $\pi_{j}$ represents the probability of category j, where j is any category other than the reference category. $\pi_{j}*$ represents the probability of the reference category “Feeding”. $\beta_{0j}$ represents the intercept parameter for category j, indicating the log odds of category j relative to the reference category “Feeding.”

#### 2.3.2 Final Model

(2) $$\ln\frac{\left(\pi_{\mathrm{ij}}\right)}{\left(\pi_{\mathrm{ij}}*\right)}=x_{i}^{T}\beta_{j},j\neq j^{*}$$

where, $\pi_{\mathrm{ij}}$ represents the probability of behavior category j given the level of disturbance x_i_.

$\pi_{\mathrm{ij}}*$ represents the probability of the reference category $j^{*}$ (feeding) given the level of disturbance x_i_. $x_{i}^{T}$ represents the vector of predictors (level of disturbance) for observation i. $\beta_{j}$ represents the vector of coefficients for behavior category j.

## 3 Results

**FIGURE 2:**
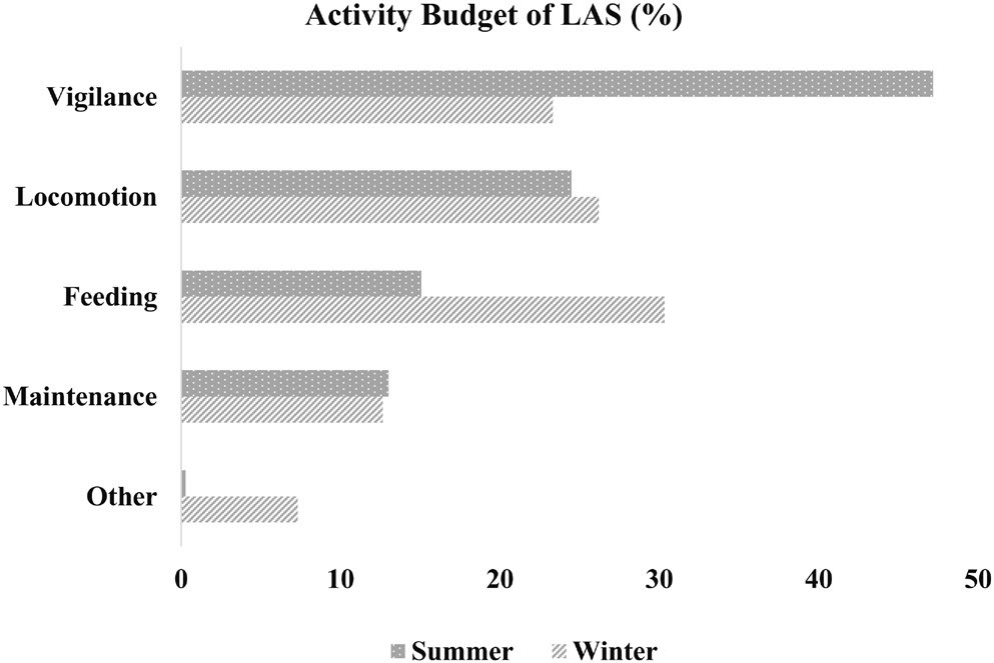
Activity budget of LAS during summer and winter.

The summer activity budget of LAS indicates a strong emphasis on vigilance behavior, which constitutes 36.41% of the total observed time. This is followed by locomotion at 33.59%, feeding at 15.90%, maintenance at 13.76%, and a minimal 0.33% spent on other activities (Figure 2). In contrast, winter observations reveal a more balanced pattern, with feeding and locomotion accounting for 28.22% and 28.42%, respectively. Vigilance behavior comprises 23.26%, while maintenance and other activities represent 11.63% and 8.49%. Overall, the seasonal comparison highlights a shift in behavior, with greater vigilance and locomotion during summer and a more evenly distributed activity budget in winter.

The descriptive statistics (Table 2) highlighted seasonal differences in stork behaviors. During Summer, the mean time spent on Feeding was 6.06 (SD = 7.89), while in Winter, it increased to 8.46 (SD = 9.25). Vigilance behavior also exhibited seasonal variation, with a mean of 9.82 (SD = 20.04) in Summer compared to 11.02 (SD = 24.61) in Winter. Locomotion was observed more in Summer (mean = 18.19, SD = 34.45) than in Winter (mean = 12.55, SD = 11.79). Maintenance behaviors were slightly higher in Winter (mean = 22.62, SD = 41.81) than in Summer (mean = 18.24, SD = 17.50). The total duration of behavioral activities varied seasonally. In summer, LAS spent the most time on locomotion (7256 s) and vigilance (7864 s), whereas in winter, feeding (4063 s) and locomotion (4092) dominated. This suggests that summer activity was influenced by increased disturbance, requiring more movement and alertness, while winter behavior of LAS reflected greater foraging effort, likely due to lower food availability.

**TABLE 2:** Association between different behaviors of LAS in different seasons.

S.N.	Functional category	Season	Duration	Mean duration	Standard deviation	No. of observation
1	Feeding	Summer	3435	6.058201	7.892283	567
2	Locomotion	Summer	7256	18.18546	34.45412	399
3	Maintenance	Summer	2973	18.23926	17.4988	163
4	Vigilance	Summer	7864	9.817728	20.03738	801
5	Feeding	Winter	4063	8.464583	9.251914	480
6	Locomotion	Winter	4092	12.55215	11.78664	326
7	Maintenance	Winter	1674	22.62162	41.81063	74
8	Vigilance	Winter	3349	11.01645	24.61203	304

The Kruskal–Wallis test results indicate that the time spent on vigilance and feeding behaviors varied significantly across seasons (*p* < 0.05), whereas Locomotion and Maintenance did not show significant seasonal differences (*p* > 0.05, Table 3). Specifically, Feeding behavior exhibited the most pronounced seasonal effect (*χ* ^2^ = 79.75, *p* value < 0.05), followed by Vigilance behavior (*χ* ^2^ = 10.99, *p* value < 0.05). In contrast, Locomotion (*χ* ^2^ = 0.55, *p* = 0.46) and Maintenance (*χ* ^2^ = 0.20, *p* = 0.65) did not show significant seasonal variations.

**TABLE 3:** Comparison of functional categories.

Functional category	Kruskal–Wallis statistic	*p*
Vigilance	10.99191	< 0.05
Locomotion	0.551891	0.457546
Feeding	79.75307	< 0.05
Maintenance	0.201745	0.653316

To further explore the significant seasonal variations detected in Vigilance and Feeding behaviors, Dunn's post hoc test was conducted. The results of Dunn's post hoc test revealed significant seasonal differences in time allocation for both vigilance and feeding behaviors (Table 4). For vigilance, a statistically significant difference was observed between summer and winter (*Z* = −3.32, *p* value < 0.05), with animals spending less time on vigilance during summer compared to winter. Similarly, feeding behavior also exhibited a highly significant seasonal variation (*Z* = −8.93, *p* value < 0.05), with individuals allocating less time to feeding in summer than in winter. These findings suggest that seasonal changes have a pronounced impact on time budgeting, particularly for critical activities such as vigilance and feeding, potentially reflecting adaptations to environmental or resource availability fluctuations between seasons.

**TABLE 4:** Seasonal Comparison of Functional Categories.

Functional category	Comparison	*Z*	*p*
Vigilance	Summer–Winter	−3.31541	< 0.05
Feeding	Summer–Winter	−8.93046	< 0.05

The Likelihood Ratio Test (LRT) comparing the full model with the null model (intercept‐only) demonstrated a statistically significant improvement in model fit upon the inclusion of the Level of Disturbance variable (LRT statistic = 263.82, df = 6, *p* < 0.05; Table 5). This indicates that disturbance levels have a meaningful effect on the behavioral variation of Lesser Adjutant Storks.

**TABLE 5:** Model comparison.

Model	Resid. Df	Resid. Dev	Test	Df	LR stat.	*p*
1	9339	7906.314				
2	9333	7642.498	1 vs. 2	6	263.8157	< 0.05

To complement the LRT and assess the statistical significance of individual model coefficients, we also conducted Wald *Z*‐tests for each behavioral category.

For locomotion, the Wald *Z*‐test produced a *Z*‐score of −2.14 for the intercept, −1.22 for low disturbance, and 0.14 for medium disturbance. The corresponding *p* values were 0.032 for the intercept, 0.224 for low disturbance, and 0.885 for medium disturbance. The significant *p* value for the intercept suggests that there are baseline factors influencing locomotion, but the disturbance levels (both low and medium) did not significantly affect locomotion behavior, as evidenced by the nonsignificant *p* values (Table 6). This indicates that locomotion appears to be insensitive to the level of disturbance in the environment.

**TABLE 6:** Effects of disturbance levels on bird behavior.

	*Z*‐sore (Intercept)	*Z*‐sore (Low disturbance)	*Z*‐sore (Medium disturbance)	*p* (Intercept)	*p* (Low disturbance)	*p* (Medium disturbance)
Locomotion	−2.14	−1.21	0.14	0.03	0.22	0.88
Maintenance	−7.61	−1.60	−0.75	< 0.05	0.10	0.44
Vigilance	11.88	−13.88	−5.25	< 0.05	< 0.05	< 0.05

In the case of maintenance behavior, the Wald *Z*‐test produced *Z*‐scores of −7.61 for the intercept, −1.61 for low disturbance, and −0.76 for medium disturbance. The *p* values for these estimates were extremely low for the intercept (*p* < 0.001), indicating that baseline maintenance behavior is highly significant. However, the *p* values for both low disturbance (0.108) and medium disturbance (0.449) were greater than 0.05, indicating that disturbance levels did not significantly influence maintenance behavior (Table 6). This suggests that the maintenance behavior of Lesser Adjutant Storks is not strongly affected by changes in disturbance levels.

For vigilance behavior, the Wald *Z*‐test revealed *Z*‐scores of 11.88 for the intercept, −13.89 for low disturbance, and −5.26 for medium disturbance. The corresponding *p* values for the intercept, low disturbance, and medium disturbance were all less than 0.05. These results indicate that both low and medium disturbance levels significantly impacted vigilance behavior, with disturbance leading to a marked decrease in vigilance as indicated by the very low *p* values (Table 6). This suggests that disturbance has a strong and negative effect on vigilance behavior, making it a highly responsive trait to environmental changes.

Together, the LRT and Wald *Z*‐test results support the validity of the overall model, confirming its robustness in explaining the relationship between disturbance levels and stork behavior.

To investigate the effect of disturbance levels on stork behavior, we applied multinomial logistic regression, with “Feeding” as the reference category for behavior, and “High” disturbance served as the reference category for the predictor variable. Therefore, all reported odds ratios for “Low” and “Medium” disturbance levels represent comparisons relative to the “High” disturbance condition.

Compared to “Feeding” under high disturbance, storks exposed to low disturbance were approximately 0.85 times more likely to engage in “Locomotion,” while those under medium disturbance were 1.03 times more likely. However, neither of these odds ratios was statistically significant (*p* > 0.05), suggesting that disturbance level had no strong influence on “Locomotion” behavior (Table 7).

**TABLE 7:** Effect of disturbance on LAS behavior.

		Level of disturbance (Ref high)								
		Coeff (Intercept)	Coeff (Low)	Coeff (Medium)	Exp coeff (Intercept)	Exp coeff (Low)	Exp coeff (Medium)	*p* (Intercept)	*p* (Low)	*p* (Medium)
Functional Behavior (Ref Feeding)	Locomotion	−0.25	−0.15	0.03	0.77	0.85	1.03	0.03	0.22	0.88
	Maintenance	−1.24	−0.29	−0.24	0.28	0.74	0.78	< 0.05	0.1	0.44
	Vigilance	1.06	−1.46	−0.98	2.9	0.23	0.37	< 0.05	< 0.05	< 0.05

For “Maintenance” behavior, storks under low and medium disturbance were 0.74 and 0.78 times as likely, respectively, to exhibit this behavior compared to feeding under high disturbance. Again, both associations were not statistically significant (*p* > 0.05), indicating no clear effect of disturbance level on maintenance behavior (Table 7).

In contrast, “Vigilance” behavior was strongly affected by disturbance level. Storks exposed to low disturbance were 0.23 times as likely, and those under medium disturbance were 0.37 times as likely to engage in vigilance compared to feeding under high disturbance. Both of these effects were statistically significant (*p* < 0.05), indicating that lower disturbance levels were associated with a significantly reduced likelihood of vigilance behavior.

## 4 Discussion

Birds allocate their time across various behaviors to ensure they gather sufficient energy for survival (Caraco 1979). These activity time budgets often vary across seasons in response to changes in environmental factors such as habitat structure, food availability, and climatic conditions (Turner 1982; Yang et al. 2007). In the present study, the vigilance behavior of LAS showed clear seasonal variation.

During summer, vigilance was recorded 801 times, with a total duration of 7864 s, resulting in an average duration of 20.04 s per event. In contrast, during winter, there were only 304 observations of vigilance, but with a total duration of 3349 s, giving a higher average of 24.61 s per event. This suggests that in summer, LAS displayed vigilance more frequently but in shorter bouts, potentially as a response to increased human disturbances such as livestock grazing, prey collection, and agricultural activity. On the other hand, vigilance events were less frequent but longer in winter, likely reflecting increased scanning effort due to scarcer food resources.

Importantly, despite the greater frequency and total vigilance time in summer, statistical analysis revealed that vigilance behavior was significantly higher in winter (*p* < 0.05), indicating that LAS allocated more intensive attention to their surroundings during each vigilance event in winter. Significant seasonal differences were observed in both feeding and vigilance behaviors of LAS, with individuals spending more time on these activities during winter compared to summer (Table 6).

This may reflect a behavioral adaptation to seasonal factors such as food scarcity and the demands of chick‐rearing, prompting storks to engage in prolonged vigilance to search for food and monitor potential threats. These findings suggest a significant seasonal shift in vigilance behavior, influenced by both environmental disturbances and ecological needs. Findings from a study on wintering Red‐crowned Crane (Wang et al. 2011) also reported similar time allocation to being vigilant, supporting the observed pattern in LAS.

Contrary to these findings, species such as the Wooly‐necked Stork (Ghimire et al. 2021) and Wattled Crane (Tadele et al. 2022) spend more time feeding during the summer compared to winter, unlike LAS. Further research is needed to understand how changes in crops and seasons affect LAS foraging behavior, as there is limited knowledge regarding the relationship between different bird behaviors (Sundar et al. 2019).

Water birds, including LAS, may alter their behavior in response to threats from predators or human disturbances, shifting from energy‐procuring activities like foraging to energy‐depleting behaviors such as vigilance or evasive movements (Schummer and Eddleman 2003; Zimmer et al. 2011; Li et al. 2015; Middleton et al. 2018). Studies indicate that water birds benefit from high‐quality habitats where prey is more readily available, leading to increased foraging efficiency and better breeding success (Janiszewski et al. 2014; Osborn et al. 2017; Haig et al. 2019). LAS, in particular, tend to nest in areas with minimal human disturbance, such as locations free from human presence and livestock grazing (Bhattarai and Kindlmann 2012). However, their preferred foraging grounds and wetlands are increasingly threatened and becoming less suitable due to escalating human activities, including the collection of prey species like fish and mollusks (Baral 2005; Sundar et al. 2016).

It reveals a clear association between varying degrees of human interference and the vigilance behavior of LAS. The findings indicate that LAS adjust their behavior according to the intensity of human disturbance, with increased emphasis on vigilance as disturbance levels rise. Specifically, LAS allocates more time to feeding activities under low disturbance conditions but prioritizes vigilance over feeding when disturbance levels are high. While other species such as the Wooly‐necked Stork have shown less vigilance near farmers indicating possible habituation to human presence (Ghimire et al. 2021), LAS remains more sensitive. Overall, these findings underscore the significant impact of disturbance on stork behavior, emphasizing the importance of considering such factors in conservation and management strategies (Bhattarai and Kindlmann 2012; Sundar et al. 2016).

## 5 Conclusion

This study offers important insights into how the LAS, a globally vulnerable wetland species, adjusts its behavior in response to seasonal variation and varying levels of human disturbance. Through systematic behavioral observations and rigorous statistical analyses—including Kruskal‐Wallis tests, likelihood ratio tests, and multinomial logistic regression, we identified vigilance as the most sensitive behavioral category to both anthropogenic pressures.

Seasonal analysis revealed a complex pattern of vigilance behavior. During summer, storks exhibited vigilance more frequently, but with shorter durations. This pattern likely reflects increased exposure to human activities, such as agricultural activities and the presence of people in foraging areas, which may trigger repeated but brief vigilance responses. In contrast, during winter, the frequency of vigilance decreased, but the duration of each vigilance bout increased significantly. This shift may be attributed to heightened energetic demands associated with lower prey availability and potential reproductive activities such as chick attendance, requiring more sustained monitoring of the environment.

Despite higher vigilance frequency in summer, statistically significant differences in vigilance behavior were found in winter, suggesting that winter presents a more ecologically demanding context, where prolonged vigilance bouts are behaviorally and energetically more consequential. This nuanced seasonal variation underscores the species' behavioral flexibility and context‐dependent strategies for coping with external stressors.

Multinomial logistic regression further supported the conclusion that disturbance levels play a key role in shaping behavioral responses, particularly vigilance. The odds of exhibiting vigilance behavior were significantly lower in areas with low and medium human disturbance, compared to highly disturbed sites. This finding affirms the utility of vigilance as a sensitive, noninvasive indicator of environmental stress.

The findings carry important conservation implications. As a vulnerable species reliant on wetland ecosystems, the LAS's behavioral adaptability, particularly in terms of vigilance can inform management decisions. Conservation efforts should prioritize reducing human disturbances, especially during breeding and foraging periods, to help safeguard critical behaviors and ensure the species' long‐term viability in rapidly changing landscapes.

In conclusion, the LAS's vigilance behavior provides a valuable behavioral lens through which to assess environmental pressures. Integrating behavioral ecology into conservation planning will be critical for the effective protection and management of this vulnerable species and its wetland habitats.

## Author Contributions

**Santosh Bajagain:** conceptualization (lead), data curation (lead), formal analysis (equal), methodology (lead), visualization (equal), writing – original draft (lead), writing – review and editing (equal). **Jhamak Bahadur Karki:** formal analysis (equal), methodology (lead), writing – review and editing (equal). **Yajna Prasad Timilsina:** formal analysis (equal), writing – review and editing (equal). **Menuka Maharjan:** formal analysis (equal), methodology (equal), writing – original draft (lead), writing – review and editing (equal). **Aavas Pradhan:** conceptualization (supporting), formal analysis (equal), methodology (equal), visualization (supporting), writing – original draft (supporting), writing – review and editing (equal). **Nabaraj Pudasaini:** writing – review and editing (equal). **Prashant Rokka:** data curation (supporting), methodology (supporting), writing – review and editing (equal). **Surendra Maharjan:** visualization (supporting), writing – review and editing (equal).

## Conflicts of Interest

The authors declare no conflicts of interest.

## Data Availability

Data will be provided upon request.
