# Correction to “Effect of Human Disturbance on Feeding Behavior and Activity Time Budget of Lesser Adjutant Stork *Leptoptilos javanicus* (Horsfield, 1821) in Nepal”

**DOI:** 10.1002/ece3.71786

**Published:** 2025-07-07

Bajagain, S., J. B. Karki, Y. P. Timilsina, et al. 2025. “Effect of Human Disturbance on Feeding Behavior and Activity Time Budget of Lesser Adjutant Stork *Leptoptilos javanicus* (Horsfield, 1821) in Nepal.” *Ecology and Evolution* 15, no. 7: e71643. https://doi.org/10.1002/ece3.71643.

**Correction to author affiliations:**

The published version of this article contains an incorrect affiliation order for author Menuka Maharjan and incorrect affiliation numbering for several authors.

The correct author list and affiliation mapping are as follows:

**Authors (corrected):**

Santosh Bajagain^1,2^ | Jhamak Bahadur Karki^3^ | Yajna Prasad Timilsina^4^ | Menuka Maharjan^2 5^ | Aavas Pradhan^1,2^ | Nabaraj Pudasaini^6^ | Prashant Rokka^7^ | Surendra Maharjan^8^

Affiliations (corrected):

^1^ Bird Conservation Nepal, Kathmandu, Nepal

^2^ School of Forestry and Natural Resource Management, Kathmandu, Nepal

^3^ Kathmandu Forestry College, Kathmandu, Nepal

^4^ Institute of Forestry, Tribhuvan University, Pokhara, Kaski, Nepal

^5^ Institute of Forestry, Tribhuvan University, Hetauda, Makwanpur, Nepal

^6^ Ministry of Forests and Environment, Kathmandu, Nepal

^7^ Faculty of Forestry, Agriculture and Forestry University, Hetauda, Makwanpur, Nepal

^8^ Chapman University, Orange, California, USA

**Correction to Abstract:**

The following line in the abstract needs to be corrected:

**Original:**

*The analysis revealed significant seasonal shifts. During the summer, vigilance dominated the activity budget (47.18%), while feeding was comparatively low (15.06%). In contrast, during winter, storks prioritized feeding (30.33%) over vigilance (23.32%)*.

**Corrected:**

*The analysis revealed significant seasonal shifts. During the summer, vigilance dominated the activity budget (36.41%), while feeding was comparatively low (15.90%). In contrast, during winter, storks prioritized feeding (28.22%) over vigilance (23.26%)*.

**Correction to Table 1:**

The descriptions in **Table 1: Functional Categories of LAS Behaviour** require revision and proper citation. The published descriptions were preliminary and have now been refined and standardized. The correct Table 1 is displayed below:

**TABLE 1:** Functional Categories of LAS Behavior. **Description (Modified from Ghimire et al. 2020 [unpublished manuscript])**.

Functional category	Behavior	Description (Modified from Ghimire et al. (2020, unpublished))
Locomotion	Walking	The bird flaps its wings when it moves. Walking occasionally causes the neck to stretch and retract.
	Leaping	Jumping short distances with one leg stretched first. Steps are separated by a greater distance than when walking.
	Running	Birds move more quickly than they walk, mostly when they are taking off, when a potential predator is approaching, or when they are feeding to capture swift‐moving prey like snakes.
	Flying	Start with quick, short wing movements. Once it reaches a certain height, the wings are steady enough to glide. It's also typical to glide before hitting the ground. Rare acrobatic manoeuvres are also displayed by the bird when it dives from a height to reach a feeding ground, roosting tree, or nest.
Maintenance	Auto preening	The bird cleans and smoothes its own wings, especially the legs, breast, belly, and flanks, with its beak. The bird cleans its own back by bending its neck.
	Scratching	The bird scratches its head, neck, and underwings with one leg. The bird uses a second leg to balance its body.
	Body fluffing	The bird conceals its beak with feathers on its back and around its breasts. Seldom does this behavior involve neck stretches.
	Wing spreading	The underside of the forearm displays bright, narrow orange‐red bands, and the wings spread widely. The bird extends its body and neck, pointing its tail downward. Wings can occasionally spread partially.
	Leg stretching	While one leg balances the body, the other stretches.
	Head shaking	A horizontal head shake is made by the bird.
	Yawning	A bird typically opens its beak to yawn after foraging. There were two occasions when one of the two birds yawned after the other.
Vigilance	Stationary head	As the bird forages in wetlands or beneath dense vegetation, it extends its neck. The head scans and stretches in one direction.
	Head Movement	The bird quickly turns its head sideways and stretches its neck.
Feeding	Foraging	In search of prey, the head is lowered to the ground. Only when searching for distant prey does the neck stretch; otherwise, it retracts.
	Probing	There are two types of probing: (1) A bird walks and probes in a single location. (2) The bird is stationary and repeatedly probes the same spot.
	Food preparation	The prey is brought closer to the tip of the beak. The bird maintains the prey's position on its beak by shaking its head and slightly opening its beak. On the ground, larger prey is immobilised first.
	Eating	The bird swallows the food after raising its head a little. While larger prey is slowly consumed, smaller prey is tossed.
	Drinking	The bird dips its beak horizontally into the water, lifts its head, and swallows.

**Correction to Figure 2**

Due to a production error, the image used for Figure 2 in the published version is incorrect. The correct figure is displayed below. The results of the study are unaffected by this correction.

**FIGURE 2:**
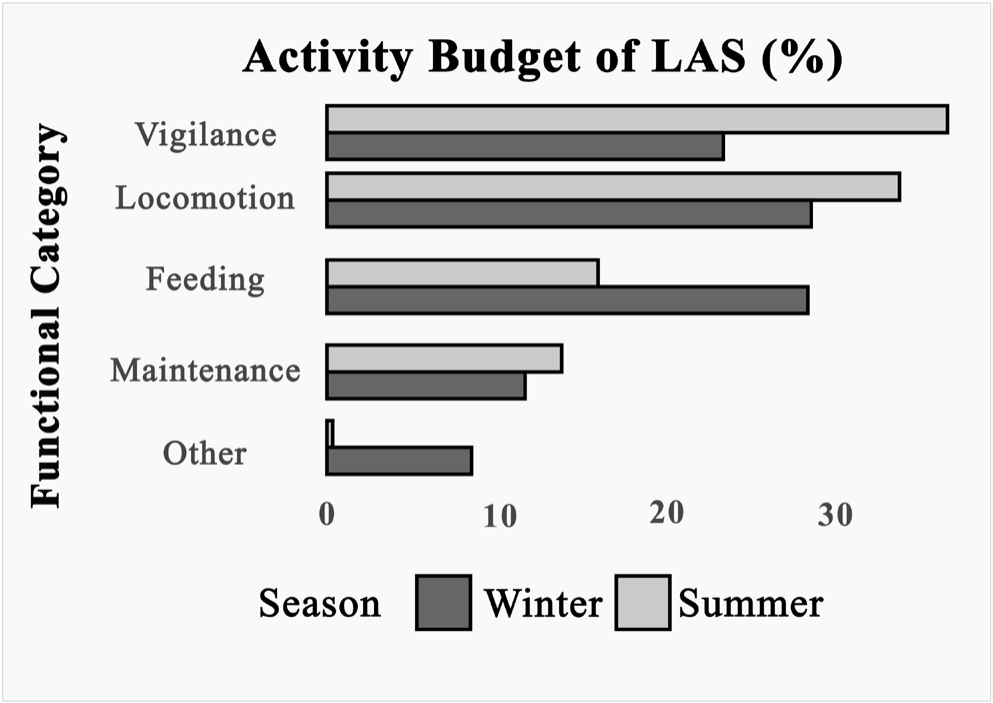
Activity budget of LAS during summer and winter.

**Reference to be added:**

Due to the corrected Table 1, a reference should be added to the article. This is displayed below:

Ghimire, P., Pandey, N., Ghimire, R., Basnet, A., Bist, B. S., Belbase, B., and Panthi, P. (2020). *An Ethology of Asian Woollyneck Ciconia episcopus* [Unpublished manuscript]. ResearchGate.

We apologize for these errors.

